# Factors Affecting Gender Differences in the Association between Health-Related Quality of Life and Metabolic Syndrome Components: Tehran Lipid and Glucose Study

**DOI:** 10.1371/journal.pone.0143167

**Published:** 2015-12-01

**Authors:** Parisa Amiri, Tina Deihim, Reza Taherian, Mehrdad Karimi, Safoora Gharibzadeh, Mohammad Asghari-Jafarabadi, Niloofar Shiva, Fereidoun Azizi

**Affiliations:** 1 Research Center for Social Determinants of Health & Obesity Research Center, Research Institute for Endocrine sciences, Shahid Beheshti University of Medical Sciences, Tehran, Iran; 2 Students' Research Committee, Shahid Beheshti University of Medical Sciences, Tehran, Iran; 3 Department of Epidemiology and Biostatistics, School of Public Health, Tehran University of Medical Sciences, Tehran, Iran; 4 Endocrine Research Center, Research Institute for Endocrine Sciences, Shahid Beheshti University of Medical Sciences, Tehran, Iran; 5 Road Traffic Injury Research Center, Tabriz University of Medical Sciences, Tabriz, Iran; Institute of Endocrinology and Metabolism, ISLAMIC REPUBLIC OF IRAN

## Abstract

**Objective:**

Using structural equation modeling, this study is one of the first efforts aimed at assessing influential factors causing gender differences in the association between health-related quality of life (HRQoL) and metabolic syndrome.

**Methods:**

A sample of 950 adults, from Tehran Lipid and Glucose Study were recruited for this cross sectional study in 2005–2007. Health-related quality of life was assessed using the Iranian version of SF-36. Metabolic syndrome components (MetSCs) and physical and mental HRQoL were considered as continuous latent constructs explaining the variances of their observed components. Structural equation modeling was performed to examine the association between the constructs of MetSCs and the physical and mental HRQoL within the two gender groups.

**Results:**

Based on the primary hypothesis, MetSCs and HRQoL were fitted in a model. The negative effect of MetSCs on HRQoL was found to be significant only in the physical domain and only in women. The proportion of all the cardio-metabolic risk factors as well as subscales of physical HRQoL that have been explained via the two constructs of MetSCs and HRQoL, respectively, were significantly higher in women. Physical activity in both men (β = 3.19, p<0.05) and women (β = 3.94, p<0.05), age (β = -3.28, p<0.05), education (β = 2.63, p<0.05) only in women and smoking (β = 2.28, p<0.05) just in men, directly affected physical HRQoL. Regarding the mental domain, physical activity (β = 3.37, p<0.05) and marital status (β = 3.44, p<0.05) in women and age (β = 2.01, p<0.05) in men were direct effective factors. Age and education in women as well as smoking in men indirectly affected physical HRQoL via MetSCs.

**Conclusion:**

Gender differences in the association between MetSCs and physical HRQoL could mostly be attributed to the different structures of both MetSCs and physical HRQoL constructs in men and women. Age and smoking are the most important socio-behavioral factors which could affect this gender-specific association in the mental domain.

## Introduction

As a chronic condition manifested by insulin resistance, central obesity, dyslipidemia, and hypertension, the metabolic syndrome (MetS) is associated with increased risk of a range of non-communicable diseases such as type 2 diabetes and cardiovascular diseases (CVDs) [1]. In addition, the relationship of MetS with poor health-related quality of life (HRQoL), especially in the physical domain is well documented [2–5], although the sex-specific pattern of this association is still unclear, our previous study and a study from Korea, both showed that MetS is associated with poor HRQoL in women, but not in men [2]. Yet another study conducted in Korea revealed a greater impairment of HRQoL in women with MetS, compared to men [3]. Levinger et al also showed that, increasing the number of metabolic risk factors was associated with lower exercise capacity, muscle strength and physical HRQoL in women, but not in men [6]. However, in a Swedish population, both men and women with MetS, showed lower levels of physical and social health [7].

To explain this gender-specific pattern some hypotheses consider psychological differences and social inequality, while others refer to different biological characteristics in men and women [8]. Findings from a previous study showed that compared to men, women with MetS experienced more pain and depression, which may be associated with psychological distress related to body shape and abdominal obesity [4]. Another study emphasized the different effects of socio-behavioral factors, e.g. educational level, physical activity, quality of sleep and doctor visits, on the personal health judgment of Swedish male and female subjects [9]; similar to these psycho-social effects, there is more evidence showing different influential patterns of cardio-metabolic risk factors on HRQoL in men and women [3]; findings similar to a previous study have demonstrated that sex-specific influential patterns of cardiovascular risk factors affect the incidence of cardiovascular outcomes [10]. Considering these psycho-biological differences, it may be possible to hypothesize that different associations between MetS and HRQoL in men and women could be due to: (i) Substantial gender differences of HRQoL and MetS as two multi-component concepts, (ii) different influential patterns of MetS components on HRQoL in each gender and (iii) different effects of socio-behavioral factors in the association between HRQoL and MetS in both genders.

Previous studies have investigated the relation between MetS and poor HRQoL using the first generation of multivariate techniques, which are limited by having to perform each analysis separately, whilst both MetS and HRQoL include several components with different effects on each other. As one of the first, the current study using a statistical approach of structural equation modeling (SEM), aimed to investigate factors affecting gender differences in the association between HRQoL and MetS components in an urban population of Tehranian adults. Using SEM allowed us to have a comprehensive view of the interaction of HRQoL with MetS components, along with an analysis of all bio-psycho-social variables simultaneously with the advantage of predicting the same standard errors and coefficients as gathered, using ordinary least square regressions.

## Materials and Methods

### Subjects and Design

This is a cross-sectional study conducted within the framework of the Tehran Lipid and Glucose Study (TLGS), between 2005 and 2007. The TLGS is an ongoing study, designed to investigate the risk factors for CVDs in an urban population of Tehran, the capital of Iran, and to implement measures at the population level, aiming at improving life styles and preventing rising trends in non-communicable diseases.

The study has two phases: The first phase (1999–2001) was a cross-sectional study and aimed at assessing the prevalence of non-communicable diseases such as diabetes and CVDs and their preceding factors; the second phase is an ongoing prospective 20-year follow-up with approximately 3-year intervals between measurements.

Primarily, all the households covered by the district’s three healthcare centers (which are officially in charge of health-related statistics in a district) were selected. To reach a similar distribution of the original population, the primary sample was stratified according to healthcare centers, following which all individuals aged < 3 years were excluded. Details on the design and rationale of the TLGS have been reported before [11].

**Fig 1:**
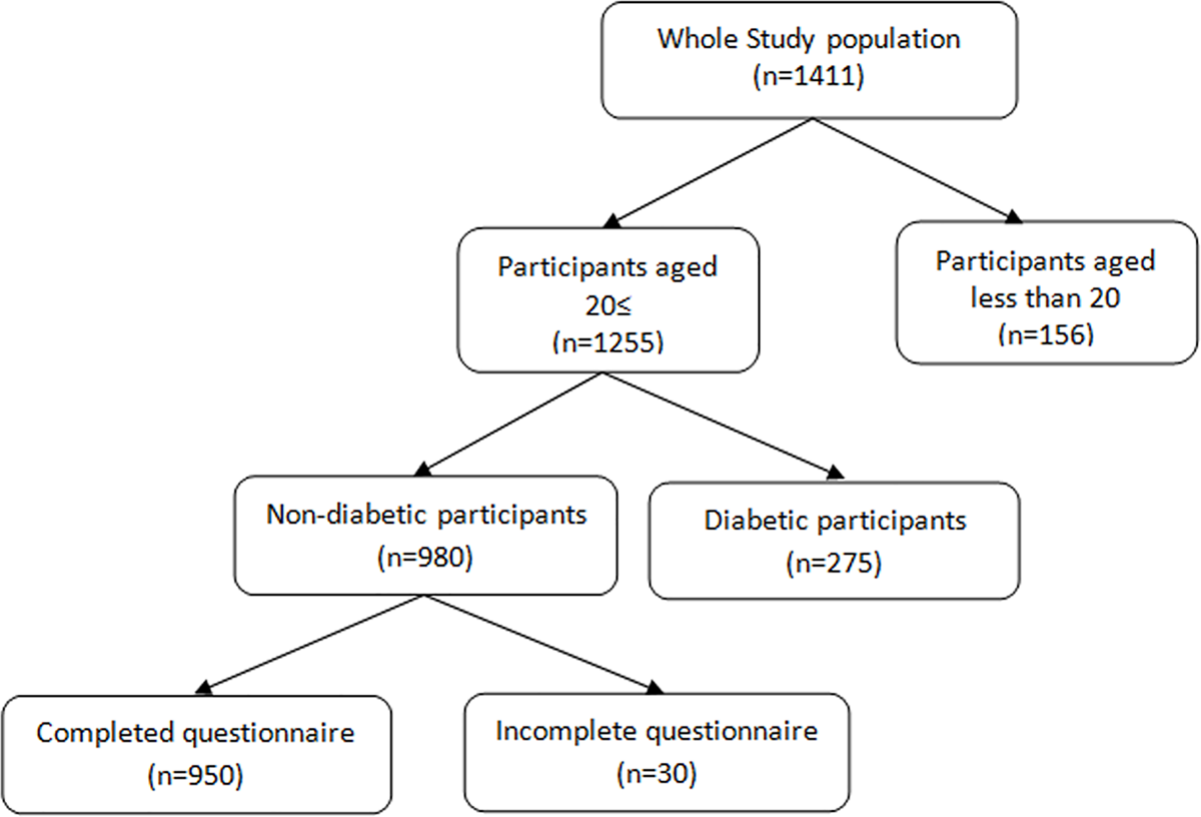
The sampling frame of the study.

For this study, Initially a sample of 1411 individuals, without any diagnosed chronic or psychological disease and using no medications, who had participated in the TLGS between September 2005 and September 2007 (phase 3), had been interviewed by a trained interviewer to collect data and had completed the SF-36 questionnaire were enrolled. Based on the study design, 1255 individuals, aged ≥ 20 years were recruited for this study, and following exclusion of 275 (21.9%) individuals with diabetes, and 30 (3.1%) individuals for missing data, left us with the data of 950 individuals (64% female) for analysis (Fig 1).

The study was approved by the ethics committee of the Research Institute for Endocrine Sciences, Shahid Beheshti University of Medical Sciences. All the participants of the study were adults and all of them provided written informed consent.

### The construct of metabolic syndrome components

Metabolic syndrome components (MetSCs) were considered as a continuous latent construct, which explains the variances of observed cardio-metabolic risk factors, including waist circumference (WC), triglycerides (TG), high density lipoprotein cholesterol (HDL-C), fasting blood sugar (FBS), systolic blood pressure (SBP) and diastolic blood pressure (DBP). The construct of MetSCs has been confirmed by the acceptable fitting indices at confirmatory factor analysis (CFA).

### Health-related quality of life

The Iranian version of the Short Form Health Survey (SF-36) measures HRQoL in the context of four physical and four mental health-related domains. Physical domains include physical functioning, role limitations attributable to physical health problems, bodily pain and general health. Mental domains include vitality, social functioning, role limitations attributable to emotional problems and mental health [12]. For each of these scales, a score of 0 and 100 was assigned as the worst and the best health conditions respectively. The reliability and validity of the Iranian version of SF-36 in the Iranian population has been reported before [13].

### Anthropometric, laboratory and behavioral measures

To measure WC to the nearest 0.1 cm, an unstretched tape meter was used at umbilical level over light clothing and recorded as the WC. Using a standard mercury sphygmomanometer, blood pressure was measured twice, first after individuals had been seated for 15 minutes and again, after at least 30s for the second time; the mean of these two measurements was documented as the individual’s blood pressure.

Blood samples were collected, after twelve-hour fasting, in tubes containing 0.1% EDTA. To separate the plasma, these blood samples were centrifuged at 4°c and 500×g for 10 min by applying an enzymatic colorimetric method with glucose oxidase. Fasting blood sugar was measured on the same day of blood collection. Using commercially available enzymatic reagents (Pars Azmoon, Tehran, Iran) adapted to a selectra autoanalyzer, serum total cholesterol and TG were measured. High density lipoprotein-cholesterol was measured after precipitation of the apolipoprotein B-containing lipoproteins with phosphotungistic acid. Low density lipoprotein-cholesterol was calculated from serum total cholesterol, TG, and HDL-C, except when TG concentration was > 4.52 mmol/L. Smoking status was considered in two groups; 1) non- and ex- smokers and, 2) current smokers. Data on age, physical activity [14] and being under treatment with oral hypoglycemic, lipid lowering or anti-hypertensive agents were obtained from TLGS data.

### Statistical analysis

First we compared the demographic and clinical variables between genders separately, using t–test and χ2 test for continuous and categorical variables respectively. Structural equation modeling was used to examine the associations among the general characteristics, MetSCs, physical and mental HRQoL. Structural equation modeling was presented in graphical forms, in two parts, the measurement model and the structural model. Relations between manifest or observed variables and latent variables are referred to as the ‘measurement model’ on a CFA and inter-relations between latent constructs are referred to as the "structural model", known as SEM.

In this study the analysis was conducted in two stages. The first stage (CFA) was used to evaluate and modify the primary constructs of MetSCs and HRQoL, both physical and mental. The structural modeling, as the second stage, was applied to reveal relations among the constructs and also to examine specific hypotheses about gender differences in this regard. By adjusting and entering the demographic variables in the structural model, we ran the adjusted SEM for testing the relations between structures and demographic variables for men and women separately. Using the χ2 difference test, various constraints in each model were checked and model comparisons were done, based on their constraints. Each model was estimated by the Maximum Likelihood Approach and compared between men and women, using multiple group analysis.

Model fit measures were obtained to assess how well the proposed model captured the covariance between all the measures. Since the sample size affects the quality of the fit models, we examined several model fit indices, which include χ2, the Root Mean Square Error of Approximation (RMSEA), the Comparative Fit Index (CFI), the ratio of the χ2 to degrees of freedom (CMIN / DF), the normed fit index (NFI), goodness of fit index (GFI) and the incremental fit index (IFI). CFI ≥ 0.90, RMSEA and SRMR ≤ 0.10 and CMIN/DF < 4.0 are considered to represent an appropriatemodel fit to the data. For GFI, IFI and NFI, which range from 0 to 1.0, values > 0.90 show an appropriate model fit to the data. IBM SPSS statistics and IBM SPSS AMOS 20 were utilized as statistical software and P values <0.05 were considered significant.

## Results

### General characteristics

Overall, 950 individuals (46.9% with MetS), aged≥20 years, were included in the study. Baseline characteristics for men and women are shown in Table 1. The mean age (SD) of participants was 46.54 (14.41) years. Significant mean differences between men and women were seen in all of the cardio-metabolic risk factors and HRQoL variables (P<0.05), except for age and physical activity. Means of all of the cardio-metabolic risk factors were higher in men than in women, except for HDL-C and BMI (p<0.05).

**Table 1:** General characteristics of study participants and their SF-36 scores.

	All (n = 950)	Men (n = 339)	Women (n = 611)	P-value
**Age (y)**	46.54 (14.41)	47.32 (14.93)	46.11 (14.11)	0.22
**Education (%)**				< 0.001
*Primary*	405 (42.9)	110 (32.4)	295 (48.4)	
*Secondary*	372 (39.4)	143 (42.2)	229 (37.9)	
*Higher*	166 (17.6)	86 (25.4)	80 (13.2)	
**Marital status (%)**				< 0.001
*Married*	784 (82.5)	297 (87.6)	487 (79.7)	
*Single/Widowed/Divorced*	166 (17.5)	42 (12.4)	124 (20.3)	
**Smoking (%)**				< 0.001
*Current*	80 (8.5)	72 (21.2)	8 (1.3)	
*Ex/Never*	862 (91.5)	267 (78.8)	595 (98.7)	
**MET-h/wk**	3.7 (0.0–15.5)	4.4 (0.0–20.3)	3.5 (0.0–13.9)	0.12
**Metabolic syndrome components (MetSCs)**				
*WC (cm)*	93.9 (12.5)	96.9 (11.3)	92.2 (12.9)	< 0.001
*BMI (kg/m²)*	28.7 (5.0)	27.6 (4.7)	29.4 (29.1)	< 0.001
*TG (mmol/L)*	1.67 (1.13–2.34)	1.74 (1.20–2.50)	1.64 (1.11–2.32)	0.03
*HDL-C (mmol/L)*	1.09 (0.27)	0.99 (0.23)	1.15 (0.27)	< 0.001
*FBS (mmol/L)*	5.02 (0.52)	5.07 (0.52)	5.00 (0.52)	0.04
*SBP (mmHg)*	118.0 (18.7)	122.1 (16.9)	115.7 (19.2)	< 0.001
*DBP (mmHg)*	74.3 (10.4)	76.5 (10.4)	73.1 (10.1)	< 0.001
**SF-36 scores**				
*Physical functioning*	77.0 (22.7)	83.1 (19.9)	73.7 (23.4)	< 0.001
*Role physical*	66.8 (36.5)	74.5 (33.9)	62.5 (37.2)	< 0.001
*Bodily pain*	71.2 (19.4)	76.4 (16.3)	68.2 (20.3)	< 0.001
*General health*	63.6 (18.9)	68.4 (18.4)	61.0 (18.7)	< 0.001
*Vitality*	61.1 (20.5)	67.6 (18.6)	57.6 (20.7)	< 0.001
*Social functioning*	74.1 (23.1)	77.4 (22.1)	72.3 (23.4)	< 0.001
*Role emotional*	62.6 (40.1)	68.5 (38.8)	59.3 (40.4)	< 0.001
*Mental health*	66.9 (19.7)	71.0 (18.4)	64.5 (20.0)	< 0.001

Data are presented as mean (SD) for continuous variables, median (25 percentile-75 percentile) for TG and MET-h/wk and n (%) for categorically distributed variables.

WC, waist circumference; BMI, body mass index; TG, triglycerides; HDL-C, high density lipoprotein-cholesterol; FBS, fasting blood sugar; SBP, systolic blood pressure; DBP, diastolic blood pressure; SF-36, short form-36

*P<0*.*05* considered significant

### Confirmatory factor analysis

**Fig 2:**
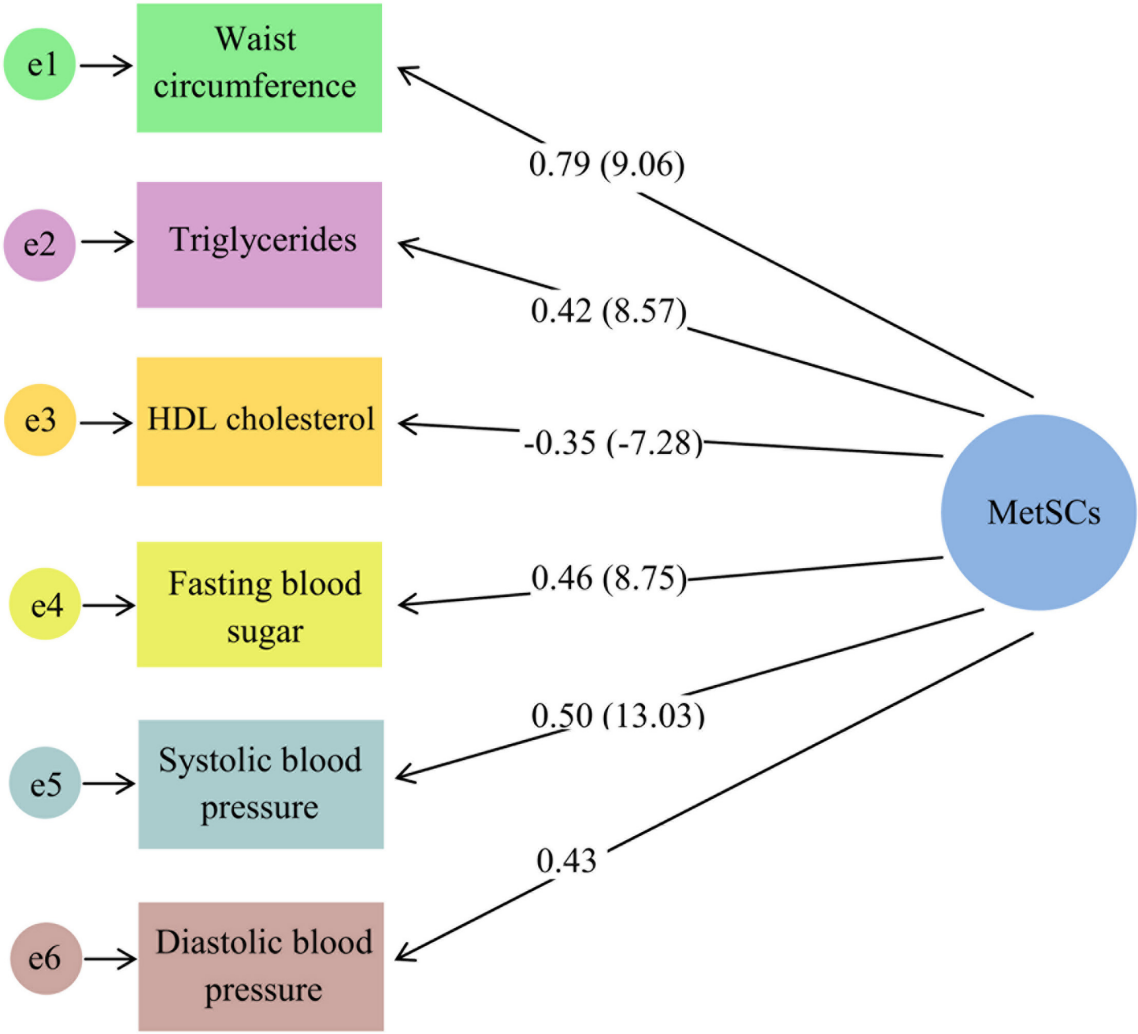
A measurement model: Construct of metabolic syndrome components (MetSCs). The numbers on these six paths represent loading factor (t-value), except for the diastolic blood pressure (fixed parameter). The model fit indices are DF = 16, CMIN/DF = 1.72, RMSEA = 0.028, RMR = 9.56, CFI = 0.99 and GFI = 0.99.

**Fig 3:**
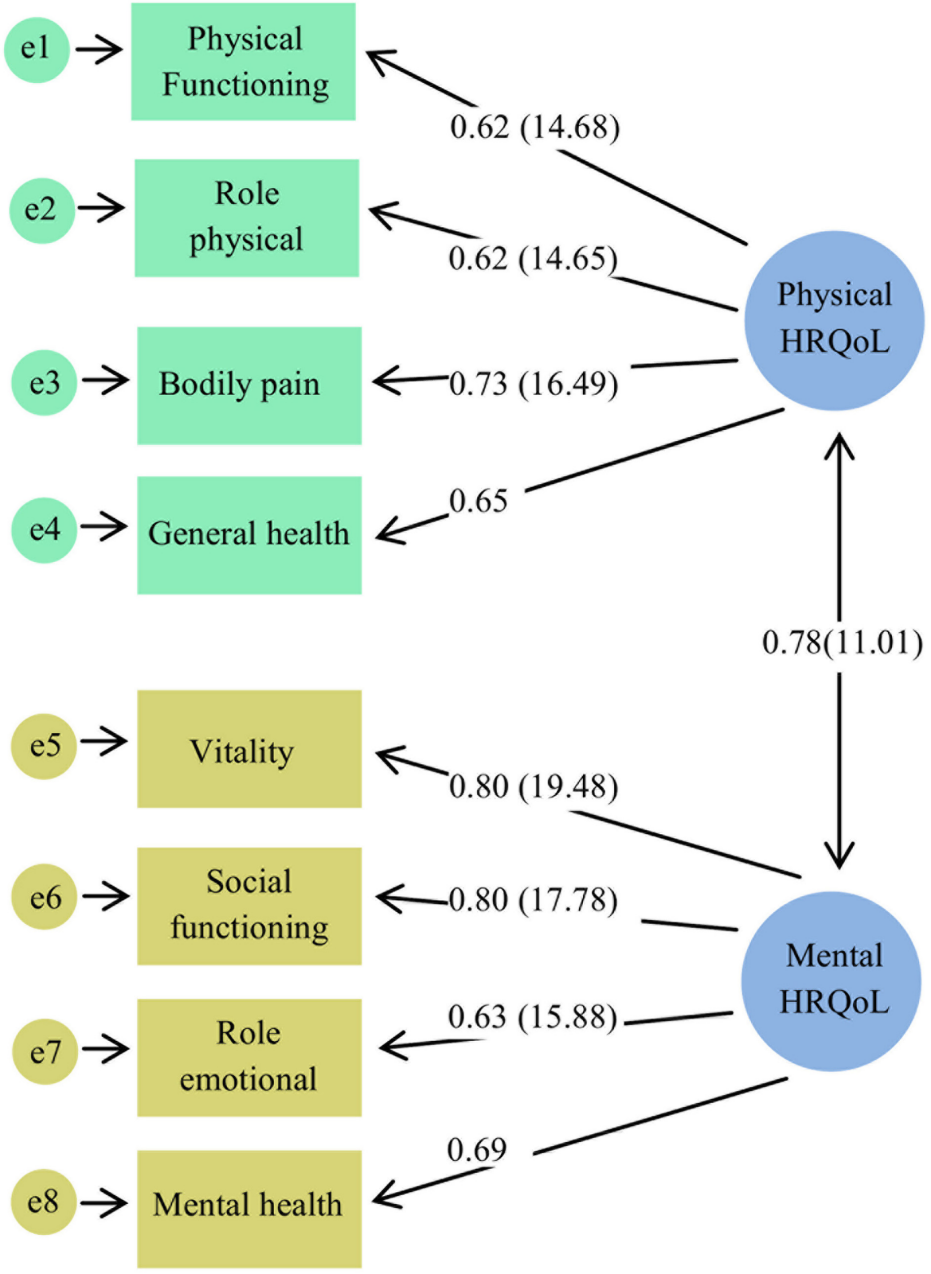
A measurement model: Construct of health-related quality of life (HRQoL) The numbers on these eight paths represent loading factor (t-value) except for general and mental health (fixed parameters). The model fit indices are DF = 26, CMIN/DF = 2.10, RMSEA = 0.034, RMR = 12.43, CFI = 0.99 and GFI = 0.99.

The fit indices for CFAs used to develop the constructs of MetSCs (χ2/df = 1.72, RMSEA = 0.028, CFI = 0.99, GFI = 0.99) and HRQoL (χ2/df = 2.10, RMSEA = 0.034, CFI = 0.99, GFI = 0.99) had acceptable fit to the data. All components were significantly related to MetSCs and HRQoL (p<0.001). Waist circumference, bodily pain and vitality explained a higher proportion of the variance of MetSCs and HRQoL, both physical and mental, constructs respectively (Figs 2 and 3).

### Structural equation modeling

**Fig 4:**
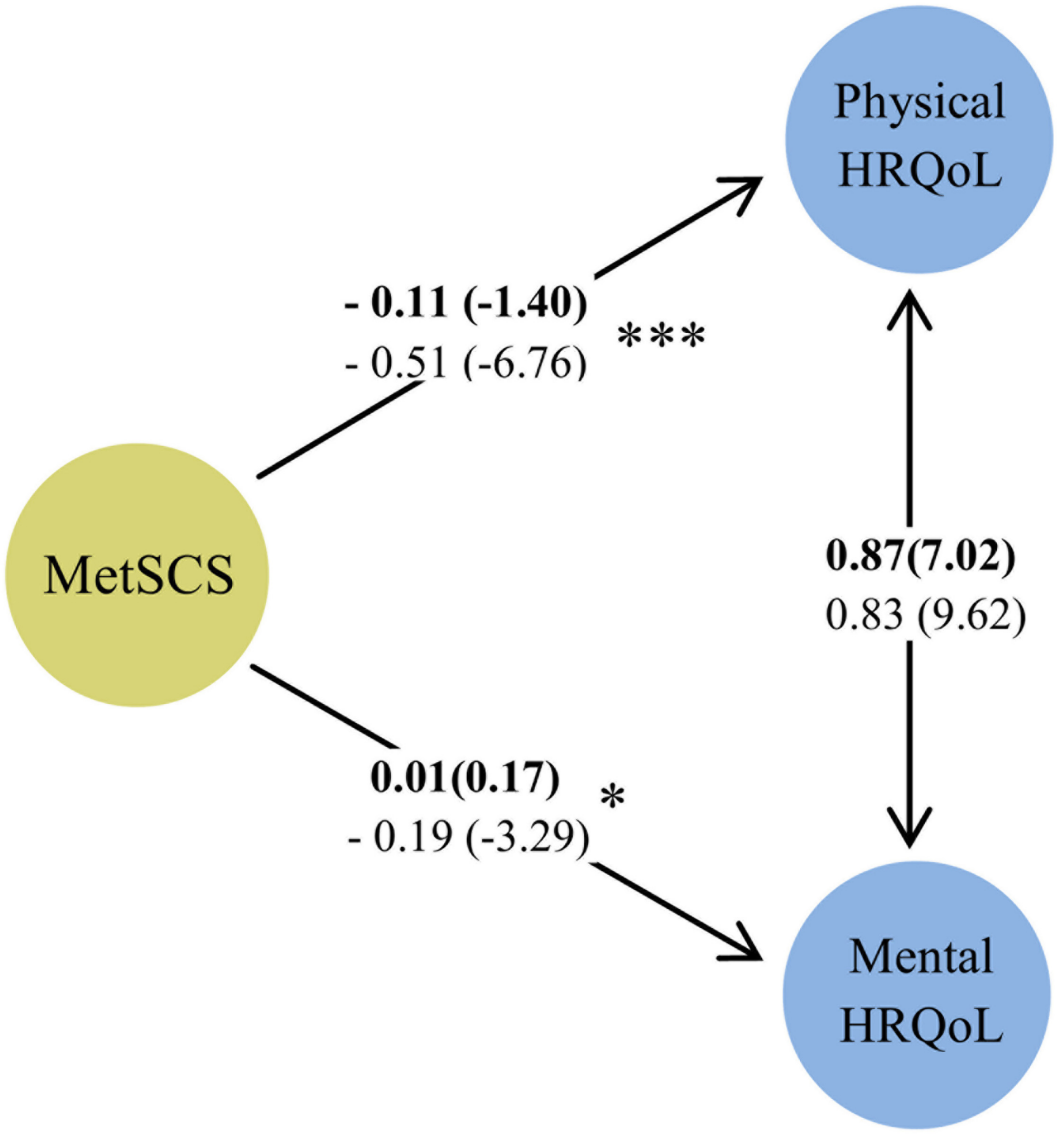
The unadjusted structural model for relations of metabolic syndrome components (MetSCs) and health related quality of life (HRQoL), both physical and mental. The numbers on the paths represent standardized regression coefficients (t-value). The two sided path shows correlation between physical and mental HRQoL. The bolded-face numbers refer to men whereas the numbers below refer to women. The significance level of the comparison of each effect between men and women is depicted by asterisks. * p<0.05, *** p<0.001.

Since the process of model fitting showed a significant modification of unconstrained over restrictive unadjusted models, all of the parameters were considered different in men and women (Table 2). Based on the primary hypothesis and suggested modification indices, the model with a correlation between physical and mental components of HRQoL, showed acceptable fitting indices (Fig 4).

**Table 2:** Alternative unadjusted models comparing the relations among metabolic syndrome components, physical and mental HRQoL.

	DF	CMIN/DF	RMSEA	RMR	CFI	GFI	NFI	IFI	Model Comparisons(CMIN,DF)
Model 1									
Unconstrained^1^	124	1.52	0.023	37.20	0.98	0.97	0.96	0.98	Assumed to be correct
Measurement weights^2^	135	1.51	0.023	39.20	0.98	0.97	0.95	0.98	14.65*, DF = 11
Structural weights^3^	137	1.61	0.025	40.66	0.98	0.97	0.95	0.98	32.25*, DF = 13
Structural covariances^4^	138	1.69	0.027	55.44	0.98	0.97	0.95	0.98	44.85*, DF = 14
Structural residuals^5^	141	1.69	0.027	59.54	0.98	0.97	0.94	0.98	49.91*, DF = 17
Measurement residuals^6^	167	1.94	0.031	128.38	0.96	0.95	0.92	0.96	135.39*, DF = 43
Model 2									
Unconstrained	226	1.99	0.032	41.99	0.96	0.95	0.92	0.96	Assumed to be correct
Measurement weights	237	2.49	0.040	58.90	0.93	0.94	0.89	0.93	140.67*, DF = 11
Structural weights	254	2.66	0.042	65.04	0.92	0.93	0.88	0.92	224.77*, DF = 28
Structural residuals	269	6.16	0.074	58.59	0.72	0.85	0.69	0.73	1206.43*, DF = 43
Measurement residual	306	5.89	0.072	105.65	0.70	0.84	0.67	0.71	1351.58*, DF = 80

* p<0.001

Model 1. Unadjusted

Model 2. Adjusted for physical activity, education, marital status, smoking and age.

DF; degree of freedom; CMIN/DF: Minimum discrepancy, divided by its degrees of freedom; RMSEA: Root mean square error of approximation; RMR: Root Mean Square Residual; CFI: Comparative-Fit index; GFI: Goodness of fit index; NFI: Normed Fit Index; IFI: Incremental Fit Index

Each constrained model compared to previous models, and their differences were tested using Chi square test. Chi square test is used to test the null hypothesis that the more constrained model is correct under the assumption that the less constrained model.

^1^ All parameters were considered different in men and women.

^2^ Equal factor loading for measurement models in men and women.

^3^ Equal factor loadings and regression weights between latent variables in men and women.

^4^ Equal covariance for independent construct (MetSCs) in men and women.

^5^ Equal residual variances for dependent constructs in men and women.

^6^All parameters were considered equal in men and women.

**Fig 5:**
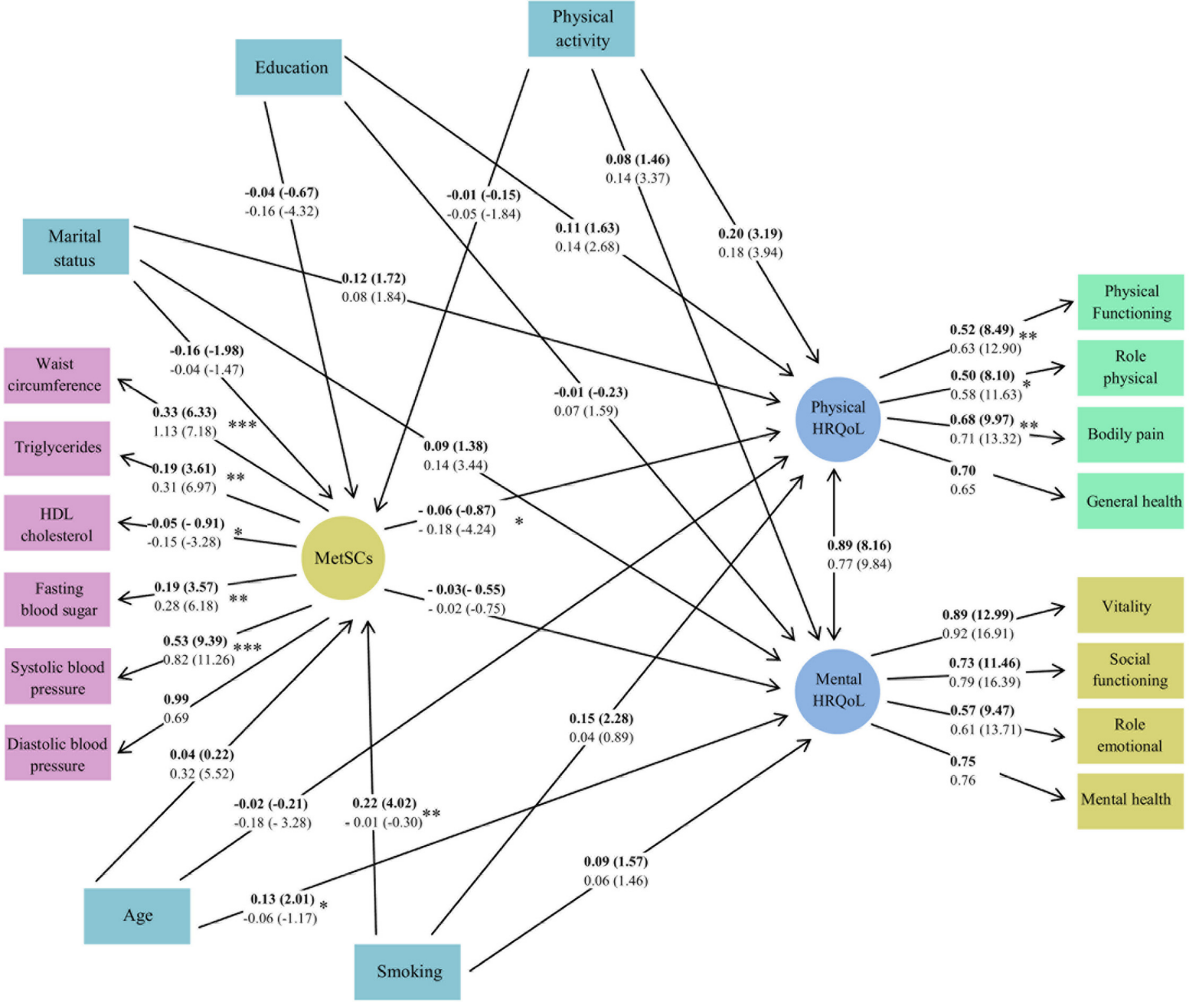
The adjusted structural model for relations of metabolic syndrome components (MetSCs) and health related quality of life (HRQoL), both physical and mental. The numbers on the paths represent standardized regression coefficients (t-value). The bold-face numbers refer to men, whereas the numbers below them refer to women. The significance level of the comparison of each effect between men and women is depicted by asterisks. * p<0.05, ** p<0.01, *** p<0.001.

Without adjusting for potential confounders, MetSCs showed a significant negative impact on both physical and mental HRQoL in women, but not in men (Fig 4). Also the correlations between the two domains of HRQoL were significant in both genders (p<0.001). However, after adjusting for potential socio-behavioral confounders, the negative effect of MetSCs on HRQoL was significant, just in the physical domain and only in women (Fig 5). Marital status and physical activity were the most important adjusting factors that affected the association between MetSCs and mental HRQoL in women.

Although in both genders, the proportions of SBP and WC as well as bodily pain were the highest among cardio-metabolic risk factors and physical subscales respectively, overall the proportion of all the cardio-metabolic risk factors as well as physical subscales were higher in women (p<0.05).

Among socio-behavioral factors, physical activity in both men (β = 0.20, p<0.01) and women (β = 0.18, p<0.001), age (β = -0.18, p<0.01) and education (β = 0.14, p<0.01) just in women and smoking (β = 0.15, p<0.05) only in men directly affected physical HRQoL. Regarding the mental domain, physical activity (β = 0.14, p<0.001) and marital status (β = 0.14, p<0.001) in women and age (β = 0.13, p<0.05) in men were direct affecting factors. In addition, among socio-behavioral factors, age and education in women had a significant effect on MetSCs, which may justify the significant effect of MetSCs on physical HRQoL in women. While age and smoking had a significant sex-specific effect on HRQoL and MetSCs, the effects of other socio-behavioral factors on HRQoL and MetSCs was not significantly different in either genders.

## Discussion

The results of the current study show a significant negative effect of MetSCs on HRQoL, just in the physical domain and only in women. Structural differences of these two multi-component constructs as well as different influential patterns of socio-behavioral factors, specifically age and smoking, seem to play a pivotal role in causing this gender difference. Although the proportions of SBP, WC and bodily pain were the highest in both genders, except for DBP, all the cardio-metabolic risk factors and physical subscales had significantly higher proportions in women.

Few studies have considered the factors contributing to the gender differences in the association between MetS and HRQoL [3, 15]. Consistent with our findings, different associations between HRQoL and abdominal obesity [16] and hypertension [17] have been previously reported in men and women. A study from Korea also revealed that, among MetS components, increased WC, only in women, and high blood pressure, in both genders, are associated with HRQoL [3]. There is more evidence regarding the relation of high TG and low HDL-C with impaired physical HRQoL [18]. However the Park et al study demonstrated that these metabolic risk factors had no significant effects on HRQoL [3]. Similarly in obese individuals, only BMI, blood pressure and FBS qualified as significant correlates of physical HRQoL [19].

The current study shows that among the components of physical HRQoL, MetSCs mostly deteriorated bodily pain and physical functioning in women. In a study from Korea, the deterioration of HRQoL by MetS in women seemed to be affected mostly by mobility and pain/discomfort [4]. There is more evidence showing that a high number of risk factors for MetS is associated with physical functioning, muscle strength and the capacity of women to perform their daily living activities, whereas this is not so in men [6]; it has also been reported that women are more likely to experience more pain at lower intensity than men [20].

Based on our results the significant impact of MetSCs on mental health in women, held no statistical significance after including socio-behavioral factors, specifically marital status and physical activity. There is much evidence contradicting the presence of a significant association between MetS and mental HRQoL [2, 5, 18]; however some studies have reported this association to be significant [21]. In line with current data, a previous study revealed the direct association between marital status and favorable well-being, especially in women [22]. Moreover marital status was reported to be stronger than age and education in explaining gender differences in mental HRQoL [23].

Previous studies have revealed a correlation between physical and mental HRQoL [24] and also the positive effect of physical health on mental health [25]. In this study, likewise, there was strong significant correlation between physical and mental HRQoL in men and women, a correlation which may yield an indirect effect of MetSCs on the mental HRQoL which is mediated by physical HRQoL, only in women. However a previous study documented a significant association between MetS and mental HRQoL [21].

In the current study, although physical activity in both genders, age and education in women and smoking in men showed significant associations with physical HRQoL, age and smoking were the most important socio-behavioral factors which could affect this gender-specific association via mental HRQoL and MetSCs respectively. The associations between socio-behavioral factors and HRQoL have been investigated by several previous studies; Morimoto et al showed that physical activity was significantly associated with physical and mental subscales of HRQoL in both genders [26], although, yet another study revealed that physical activity does not affect mental HRQoL in Brazilian women [27]. More data shows that low levels of education and low physical quality of life are positively associated with one or more of the features of MetS in women [28]. Nonetheless, Cherepanov et al reported that marital status, compared to socio-behavioral factors including education and age, explains gender differences better in physical HRQoL [23]. Our findings regarding the positive effect of smoking on MetSCs and physical HRQoL in men are inconsistent with that of a previous study revealing that smokers had significantly lower HRQoL than non-smokers [29]; however, these different effects of smoking on men and women in the current study may be due to limited numbers of female smokers. In addition, our results emphasize the positive effect of age on mental health in men, which could indirectly affect their physical HRQoL, findings in agreement with those of Mercier et al, who reported that older individuals in both genders, expressed higher satisfaction compared to their younger counterparts [30]. Moreover Lehman et al showed that women, aged 36–45 years, expressed significantly lower satisfaction of their health, compared with men in this age range [31].

To mention the strengths of our study, although previous studies revealed a sex-specific association between MetS and HRQoL, to the best of our knowledge, this is the first study considering factors affecting this difference using SEM, an appropriate analysis technique, hitherto unused in this field; however our results must be interpreted in the light of certain limitations. Because of the cross-sectional nature of the current study, it is hard to determine the causal effects. Furthermore due to data limitations, we could not enter all of the influential psycho-social factors such as depression, effective social norms, job and socio-economic status, which could have resulted in different HRQoL in both genders, in the hypothesized model. Moreover the small number of female smokers precludes a definitive statement about the effect of smoking on this gender disparity in the association of MetS with dimensions of HRQoL. In summary, the gender differences in the association between MetSCs and physical HRQoL could mostly be attributed to different structures of both MetSCs and physical HRQoL constructs in men and women. Age and smoking are the most important socio-behavioral factors which could affect this gender-specific association via mental HRQoL and MetSCs respectively. Although the current results may not show casual effects and there is also a possibility of existing other models that fit better than the current one, these results indicate the importance of gender specific programs to control CVDs and also the pivotal role of mental care to promote physical HRQoL in urban middle-aged women with cardio-metabolic risk factors. Further investigation is needed to confirm the current findings in different populations.
